# Pre-Analytical Sample Quality: Metabolite Ratios as an Intrinsic Marker for Prolonged Room Temperature Exposure of Serum Samples

**DOI:** 10.1371/journal.pone.0121495

**Published:** 2015-03-30

**Authors:** Gabriele Anton, Rory Wilson, Zhong-hao Yu, Cornelia Prehn, Sven Zukunft, Jerzy Adamski, Margit Heier, Christa Meisinger, Werner Römisch-Margl, Rui Wang-Sattler, Kristian Hveem, Bruce Wolfenbuttel, Annette Peters, Gabi Kastenmüller, Melanie Waldenberger

**Affiliations:** 1 Institute of Epidemiology II, Helmholtz Center Munich, Munich, Germany; 2 Institute of Experimental Genetics, Helmholtz Center Munich, Munich, Germany; 3 Central Hospital of Augsburg, KORA Myocardial Infarction Registry, Augsburg, Germany; 4 Institute for Bioinformatics and Systems Biology, Helmholtz Center Munich, Munich, Germany; 5 Department of Public Health and General Practice, Norwegian University of Science and Technology, Levanger, Norway; 6 Department of Endocrinology, University Medical Center Groningen, Groningen, The Netherlands; Korea University, KOREA, REPUBLIC OF

## Abstract

Advances in the “omics” field bring about the need for a high number of good quality samples. Many omics studies take advantage of biobanked samples to meet this need. Most of the laboratory errors occur in the pre-analytical phase. Therefore evidence-based standard operating procedures for the pre-analytical phase as well as markers to distinguish between ‘good’ and ‘bad’ quality samples taking into account the desired downstream analysis are urgently needed. We studied concentration changes of metabolites in serum samples due to pre-storage handling conditions as well as due to repeated freeze-thaw cycles. We collected fasting serum samples and subjected aliquots to up to four freeze-thaw cycles and to pre-storage handling delays of 12, 24 and 36 hours at room temperature (RT) and on wet and dry ice. For each treated aliquot, we quantified 127 metabolites through a targeted metabolomics approach. We found a clear signature of degradation in samples kept at RT. Storage on wet ice led to less pronounced concentration changes. 24 metabolites showed significant concentration changes at RT. In 22 of these, changes were already visible after only 12 hours of storage delay. Especially pronounced were increases in lysophosphatidylcholines and decreases in phosphatidylcholines. We showed that the ratio between the concentrations of these molecule classes could serve as a measure to distinguish between ‘good’ and ‘bad’ quality samples in our study. In contrast, we found quite stable metabolite concentrations during up to four freeze-thaw cycles. We concluded that pre-analytical RT handling of serum samples should be strictly avoided and serum samples should always be handled on wet ice or in cooling devices after centrifugation. Moreover, serum samples should be frozen at or below -80°C as soon as possible after centrifugation.

## Introduction

Due to technological advances in various omics fields, it is now feasible to measure human samples in high-throughput analysis. Research projects in areas such as genomics, proteomics and metabolomics analyze huge sample numbers to obtain reliable results. Therefore, funding organizations have spent hundreds of millions of dollars in building up large biobanks in recent years to provide access to the necessary samples for researchers [1, 2]. Nevertheless, the varying quality of the existing biospecimens in biobanks has been identified as one of the major issues inhibiting scientific progress [3]. Even before a sample is physically removed from the donor, aberrant changes to the contained biomolecules can occur; these changes can also result from all subsequent handling, processing and storage steps [4, 5]. It has been demonstrated that most laboratory errors occur in the pre-analytical phase [6]. Carefully following standard operating procedures (SOPs) can reduce the bias imposed by sample handling. Many of the currently used SOPs, however, are based on “best practices” and not on experimental findings. Studies addressing such questions under “real life” conditions are therefore urgently needed [4].

In large-scale epidemiological or clinical studies it can be difficult or even impossible to treat samples optimally, for example when transport to central laboratories is required [7, 8]. It is therefore essential that researchers know whether they can use a specific sample or collection of samples obtained from a biobank or study to address a certain research question. Specific endogenous markers for sample quality would be a great benefit to research involving biobanked samples. Another important point is that degradation rates and susceptibility to adverse handling conditions differ considerably between different biomolecules. While for example DNA is quite stable, proteins and some metabolites are rather labile and high sample handling standards have to be applied to avoid artifacts. As a consequence, quality metrics should be specific to the desired downstream analysis.

In the context of the BioSHaRE-EU-project, we conducted the study presented here. We focused on metabolomics as the downstream analysis. Metabolite concentrations provide a direct readout of biological processes in the human body and are associated with disorders such as cardiovascular and metabolic diseases [9, 10]. Due to higher metabolite concentrations, serum has been shown to be better suited for biomarker analysis than plasma [11].

We investigated the influence of up to four freeze-thaw cycles and of different pre-storage handling conditions on metabolomics parameters measured in serum, based on a targeted metabolomics approach. We present a measure that is able to distinguish between ‘good’ and ‘bad’ pre-analytical sample quality in our study.

## Material and Methods

### Study population

19 healthy male volunteers were included in the study and written informed consent was obtained from all participants. The study was approved by the “Ethikkommission der Bayerischen Landesärztekammer” and by the data security officer of the Helmholtz Center Munich. Study participants were aged between 20 and 35, were normal weight according to the German Society for Nutrition (Deutsche Gesellschaft für Ernährung: body mass index between 20 and 25) and were not taking permanent medication and no medication at all 48 hours prior to blood sampling. Subjects were examined (height, weight) and asked to provide data on their lifestyle habits (alcohol, nicotine and food consumption 24 hours prior to sampling). Of 19 participants, 7 were smokers and had consumed between 0 and 5 cigarettes in the 8 hours before sampling and 6 had consumed alcohol in the 24 hours prior to sampling. All subjects began fasting eight hours prior to blood sampling (no food, no drinks except water).

### Sample handling

Two serum gel monovettes (Sarstedt 02.1388) were drawn from each individual. Sampling took place as described in BioSHaRE SOP WP5-001-blood withdrawal (www.bioshare.eu). All relevant time points (blood withdrawal, start of centrifugation, aliquoting, etc.) were recorded in a biosample protocol for each participant and were within the ranges stated in the SOP.

After sampling, serum monovettes were stored upright for 30 min at room temperature (RT) to allow for clotting. Serum was separated at 2000 x g for 15 min at 15°C. Serum from both monovettes of each individual was pooled in precooled (ice-water) round bottom tubes and mixed.

Pooled serum of each participant was aliquoted in 13 aliquots of 100 μl in precooled cryo tubes, resulting in one aliquot for each of the freeze-thaw and handling conditions. 500 μl of serum of each subject was additionally transferred to a 15 ml tube to obtain a serum pool of all participants to be used as a technical reference. Pooled serum was mixed and 200 μl aliquots were prepared in precooled cryo tubes. When the serum pool was completed, the time was recorded as time point zero for the handling experiment. All handling steps were carried out on ice and aliquots were kept on ice until time point zero. The time span between the centrifugation of serum of the first participant and time point zero was 5 hours.

**Fig 1:**
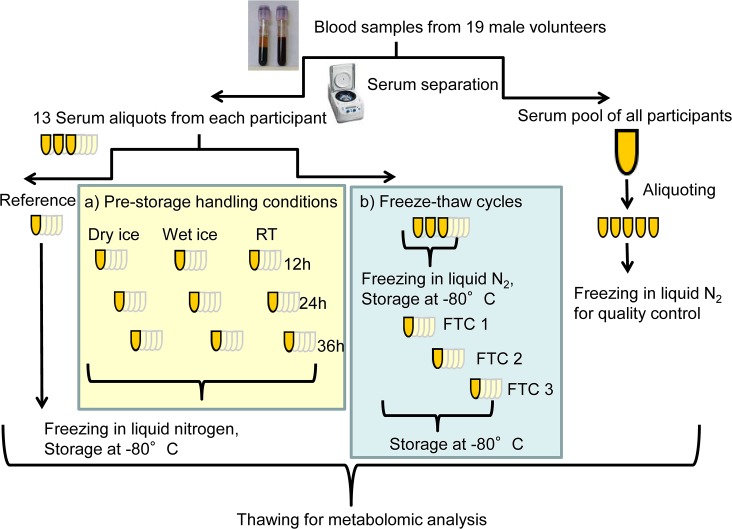
Experimental design. 13 serum aliquots from each of the 19 male volunteers and a serum pool of all participants were prepared from blood samples; one reference sample from each participant and the serum pool aliquots were immediately frozen in liquid nitrogen; a) 9 aliquots for the experiment of pre-storage handling conditions were kept at RT, on wet ice or on dry ice for 12, 24 or 36 hours and then frozen in liquid nitrogen; b) for the experiment on freeze-thaw cycles three aliquots of each participant were immediately frozen in liquid nitrogen, stored at -80°C and subjected to one to three additional freeze-thaw cycles (FTC: 1 hour thawing at RT, mixing, refreezing at -80°C). All aliquots were subsequently stored at -80°C until analysis by a targeted metabolomics approach.

All serum pool aliquots, aliquots from each participant for the freeze-thaw experiment and the reference samples from each individual were immediately frozen in liquid nitrogen and stored at -80 °C (Fig 1).

### Freeze-thaw cycles

Serum aliquots of each participant were stored at -80°C and subjected to up to four freeze-thaw cycles. A freeze-thaw cycle consisted of thawing aliquots for one hour at RT, mixing them thoroughly and setting them back to -80°C. Aliquots of each participant were either only thawed once for analysis (reference sample) or subjected to one, two or three additional freeze-thaw cycles as described above and then thawed for analysis (FTC 1, FTC 2, FTC 3) (Fig 1). The time between individual freeze-thaw cycles was one week.

### Pre-storage handling conditions

For the examination of different handling conditions before long-term storage and to simulate sample transport, serum aliquots of each participant were stored on dry ice, on wet ice or at RT; for each of the storage conditions, the serum aliquots were stored for 12, 24 or 36 hours, resulting in a total of 9 storage condition/time combinations. After the corresponding delay time, aliquots were frozen in liquid nitrogen and stored at -80°C until analysis. Reference samples were frozen in liquid nitrogen without delay and only thawed once for analysis (Fig 1). RT was recorded periodically and was between 22 and 24°C throughout the 36 hours of the experiment.

### Metabolomics measurements

The targeted metabolomics approach was based on flow injection analysis—electrospray ionization—triple quadrupole mass spectrometry (FIA-ESI-MS/MS) using the Absolute*IDQ* p150 kit (Biocrates Life Sciences AG, Innsbruck, Austria). This method allows simultaneous quantification of 163 metabolites from serum [9, 12]: 14 amino acids, hexose (sum of hexoses), free carnitine, 40 acylcarnitines (Cx:y), 15 sphingomyelins (SM(x:y)), 77 phosphatidylcholines (PC(x:y)) and 15 lysophosphatidylcholines (LPC(x:y)). Thereby, x:y denotes the number of carbon atoms (x) and the number of double bonds (y) in the fatty acid side chain(s) of the lipid. Measures for phosphatidylcholines and sphingomyelins, which contain two distinct fatty acid chains, include all isobaric PC and SM species with the same sum of carbons and sum of double bonds in the two fatty acid chains (i.e. PC(38:4) includes PC(18:1/20:3), PC(18:0/20:4), PC(16:2/22:2), etc.). The analytical specifications for the Absolute*IDQ* p150 kit regarding linearity, precision and accuracy, reproducibility and stability of metabolite quantifications are described as part of the analytical specifications document AS-P150 (Biocrates). This document can be provided by the authors upon request. The method has been proven to be in conformance with the FDA-Guideline “Guidance for Industry—Bioanalytical Method Validation” (May 2001), which implies proof of reproducibility within a given error range. In our study, sample preparation and measurements were performed as described in [12] and in the Absolute*IDQ* p150 user’s manual UM-P150. Briefly, after centrifugation, 10 μL of serum was pipetted into a 96 well sandwich plate which contained inserted filters holding stable isotope labeled internal standards. After drying the filters in a nitrogen stream, amino acids were derivatized with 5% phenylisothiocyanate reagent (PITC). Filters were dried again and metabolites and internal standards were extracted with 5 mM ammonium acetate in methanol. The solution was centrifuged through the filter membrane of the plate bottom and diluted with MS running solvent. Liquid handling was performed on a Hamilton Microlab STAR robot (Hamilton Bonaduz AG, Bonaduz, Switzerland). The prepared samples were analyzed by FIA-ESI-MS/MS on an API 4000 mass spectrometer (AB Sciex Deutschland GmbH, Darmstadt, Germany) using multiple reaction monitoring (MRM). Metabolites were quantified by reference to appropriate internal standards.

Treated subject samples were measured once each. To avoid batch effects, all samples from the same participant were analyzed on the same plate. For quality control, three zero samples (phosphate buffered saline with internal standards) and five samples of serum pool of all participants were measured on each of the four kit plates. To ensure data quality, each metabolite had to meet two criteria: (1) the coefficient of variance (CV) for the metabolite in the total of 20 pool samples had to be smaller than 25%; (2) 50% of all measured sample concentrations for the metabolite had to be above the limit of detection (LOD), which was defined as 3 times the mean of the 12 zero samples. In total, 127 metabolites passed these quality controls. A list of all 163 metabolites measured and the 127 metabolites which passed the described quality control is given in S1 Table. Concentrations of all analyzed metabolites are reported in μM.

### Statistical Analysis

All statistical analysis was performed in R version 3.1.0 (http://www.r-project.org/), with packages randomForest (version 4.6.10) and rpart (version 4.1.8).

To investigate the effects of time and temperature on metabolite concentrations we applied a mixed effects linear regression model for each metabolite, with dependent variable concentration and fixed effects time, temperature and the interaction of time and temperature, and a random effect identifying the study participant. To find significant metabolites, we used the p-values of the regression coefficients with a Bonferroni correction of p < 1.3 *10^–4^ (p < 0.05/(3*127)), due to three coefficients per model and 127 models. For the effect of freeze-thaw cycles on metabolite concentrations we used again a mixed effects linear regression model but with fixed effect number of freeze-thaw cycles and a Bonferroni correction of p < 3.9*10^–4^ (p <0.05/127), due to 127 models.

To account for individual variations in the samples, we examined the effect of time and temperature on metabolite ratios, again using a mixed effects linear regression model for each pair of metabolites, with study participant as the random effect and time and temperature as the fixed effects. The outcome used was log (concentration of metabolite 1)—log (concentration of metabolite 2) (logarithms used for ease of calculation). In addition to the p-values for the time and temperature coefficients, the ‘overall’ p-value of the model was calculated using the likelihood ratio test for the model compared to the null (intercept) model. To further obtain information on the added value of examining the ratios rather than the individual metabolites, we took advantage of the ‘p-gain’ of the temperature coefficient, time coefficient, and overall model. The p-gain is defined as the increase in the strength of association, expressed as the change in p-value when using ratios compared to the smaller of the two p-values when using the two metabolite concentrations individually [13].

Considering we ran 8001 (127*126/2) ratio models, we looked for those pairs that resulted in a p-value <2.8*10^–6^ (p <0.05/(8001*3)) and at the same time a p-gain > 240030 (p-gain >10*3*8001) [13] in either temperature, time or overall model. 235 pairs satisfied these requirements. To examine the effect of freeze-thaw cycles on metabolite ratios we looked for pairs with a p-value <6.3*10^–6^ (p <0.05/8001) and a p-gain > 80010 (p-gain >10*8001). No pair fulfilled these demands.

Our ultimate goal was to find an intrinsic measure to distinguish ‘good’ from ‘bad’ pre-analytical quality samples. With the changes in single metabolite concentrations and metabolite concentration ratios we had seen, we were able to define two sets of ‘good’ and ‘bad’ quality samples in order to perform supervised classification: a stringent criteria set which defines the reference samples which had been frozen in liquid nitrogen immediately after serum preparation as ‘good’ quality samples, and samples kept for 36 hours at RT as ‘bad’ quality samples; and a non-stringent criteria set, which additionally defined all samples kept on dry ice as ‘good’ quality samples, and samples kept for 24 hours at RT as ‘bad’ quality samples. Additionally, we divided our data set into a training set and a test set by randomly selecting 12 participants (Nr. 1, 4, 5, 7, 9, 11, 12, 14, 15, 16, 18, 19) for the training set and 7 (Nr. 2, 3, 6, 8, 10, 13, 17) for the test set.

We used a 2-step process for classification based on the training set. First, we determined the 10 most important variables for the distinction between ‘good’ and ‘bad’ quality samples using the stringent quality definition (‘good’: reference samples; ‘bad’: 36h at RT). Second, we constructed the classification rules based on these 10 variables and the samples under the non-stringent quality definition (‘good’: reference samples and samples from all time points on dry ice: 48 samples; ‘bad’: 24h and 36h at room temperature: 24 samples).

In the first step, we used Random Forest analysis (RF) [14] to select the most important variables. In addition to “single” metabolite ratios as variables, we considered the following sum ratios: total lysophosphatidylcholines/total phosphatidylcholines (tLyso/tPC), tLyso/total diacylphosphatidylcholines (tLyso/tPCaa), tLyso/total acylalkylphosphatidylcholines (tLyso/tPCae) and total amino acids/total acylcarnitines, resulting in a total of 239 variables. Running 500 RF (ntree = 5001, mtry = 80) iterations, we averaged the importance for each variable according to the mean decrease in classification accuracy. This gave us a ranking of importance of the 239 variables.

In the second step, we created a simple classification tree [15] based on the 10 variables selected in step one, using the R package rpart. For this step, we included the samples with the non-stringent quality definition of the 12 individuals in the training set.

Finally, we applied the resulting classification tree to the samples of the 7 individuals in the test set. Thereby, we obtained the misclassification rate of the model (under non-stringent definition of ‘good’ and ‘bad’).

To estimate the robustness of our approach, we additionally tested varying numbers and combinations of the top ten variables to create further classification trees, and determined their misclassification rates.

## Results

### Concentration changes in 24 metabolites due to room temperature handling

We examined the effects of different pre-storage handling conditions on the concentrations of the 127 single metabolites which remained after quality control (S1 Table).

Of those 127 metabolites, 24 displayed significant p-values (<1.33*10^–4^) for the temperature coefficient. Of those, 18 metabolites additionally yielded significant p-values for the interaction between temperature and time, and out of which 12 metabolites also showed significant p-values for the time coefficient (Table 1). We do not find metabolites that are only significant for the time coefficient or the interaction. This shows that the temperature is the most important factor in our study and the time only plays a role in combination with temperature.

**Table 1:** Metabolites with significant concentration changes due to the pre-storage handling conditions of the samples.

Metabolite name	Short name	Molecule class	p-value of linear regression model ^1^			Direction of change
			time	temp	time:temp	
Decadienylcarnitine	C10:2	Acylcarnitine	0.578	**2.00E-05**	0.174	-
Arginine	Arg	Amino acid	0.016	**1.05E-05**	**9.98E-05**	+
Glycine	Gly	Amino acid	1.55E-04	**2.27E-10**	**1.50E-07**	+
Ornithine	Orn	Amino acid	0.074	**5.96E-10**	**2.49E-07**	+
Phenylalanine	Phe	Amino acid	**1.17E-07**	**4.87E-14**	**1.48E-12**	+
Serine	Ser	Amino acid	**7.51E-07**	**3.58E-15**	**7.58E-14**	+
Leucine+Isoleucine	Leu+Ile	Amino acid	0.204	**6.41E-06**	0.001	+
Lysophosphatidylcholine LPC(14:0)	lysoPC(14:0)	Glycerophospholipid	**1.84E-09**	**6.32E-18**	**1.58E-24**	+
Lysophosphatidylcholine LPC(16:0)	lysoPC(16:0)	Glycerophospholipid	**3.93E-24**	**1.65E-25**	**2.94E-51**	+
Lysophosphatidylcholine LPC(16:1)	lysoPC(16:1)	Glycerophospholipid	**1.24E-09**	**3.60E-18**	**2.02E-23**	+
Lysophosphatidylcholine LPC(17:0)	lysoPC(17:0)	Glycerophospholipid	**2.32E-17**	**1.41E-21**	**1.17E-40**	+
Lysophosphatidylcholine LPC(18:0)	lysoPC(18:0)	Glycerophospholipid	**1.05E-25**	**1.09E-25**	**3.64E-53**	+
Lysophosphatidylcholine LPC(18:1)	lysoPC(18:1)	Glycerophospholipid	**3.17E-10**	**1.90E-21**	**3.39E-31**	+
Lysophosphatidylcholine LPC(20:0)	lysoPC(20:3)	Glycerophospholipid	**3.02E-05**	**8.71E-15**	**1.01E-14**	+
Lysophosphatidylcholine LPC(20:4)	lysoPC(20:4)	Glycerophospholipid	**2.85E-05**	**6.38E-14**	**3.62E-17**	+
Diacylphosphatidylcholine PC(30:0)	PC(30:0)	Glycerophospholipid	0.021	**3.00E-06**	**1.14E-05**	-
Diacylphosphatidylcholine PC(32:0)	PC(32:0)	Glycerophospholipid	0.141	**5.70E-05**	0.015	-
Diacylphosphatidylcholine PC(32:1)	PC(32:1)	Glycerophospholipid	**7.80E-10**	**7.94E-12**	**7.34E-19**	-
Diacylphosphatidylcholine PC(32:2)	PC(32:2)	Glycerophospholipid	0.002	**4.52E-08**	**3.87E-09**	-
Diacylphosphatidylcholine PC(34:2)	PC(34:2)	Glycerophospholipid	0.005	**4.72E-06**	**7.78E-05**	-
Diacylphosphatidylcholine PC(34:3)	PC(34:3)	Glycerophospholipid	**4.75E-05**	**1.04E-10**	**3.11E-11**	-
Diacylphosphatidylcholine PC(34:4)	PC(34:4)	Glycerophospholipid	0.008	**1.61E-05**	0.004	-
Diacylphosphatidylcholine PC(36:5)	PC(36:5)	Glycerophospholipid	0.009	**6.17E-05**	0.001	-
Acylalkylphosphatidylcholine PC(O-34:1)	PC(O-34:1)	Glycerophospholipid	0.086	**4.65E-05**	0.027	-

^1^: metabolites displayed significant p-values (in bold) of the linear regression model either for temperature alone (temp), for temperature and interaction (time:temp) or for time (time), temperature and interaction; the column on the right indicates the direction of the observed concentration changes.

**Fig 2:**
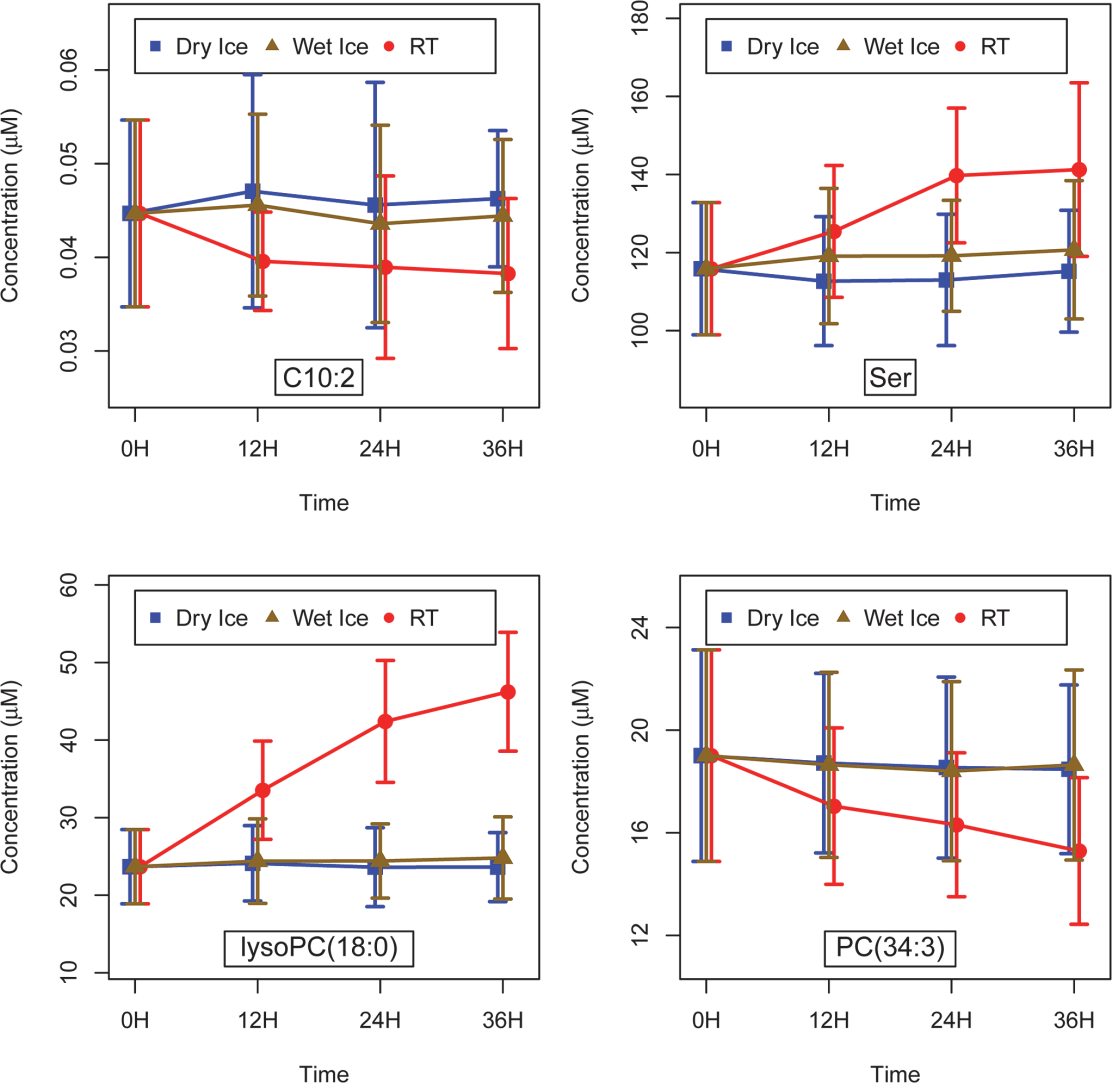
Examples of metabolite concentration changes associated to prolonged exposure of samples to room temperature in three metabolite groups. The displayed concentration values represent the mean of the samples of all 19 participants for the respective pre-analytical condition. The error bars represent one standard deviation based on the 19 measurements. Concentrations of the acylcarnitine decadienylcarnitine (C10:2), the amino acid serine (Ser), the glycerophospholipids lysophosphatidylcholine lysoPC(18:0) and diacylphosphatidylcholine PC(34:3) changed due to different pre-storage handling conditions of samples.

In the group of 21 acylcarnitines, we found only one metabolite with a significant p-value for the temperature coefficient, decadienylcarnitine (C10:2; p = 2.00*10^–5^) (Fig 2). Concentrations for C10:2 already decreased between 0 and 12 hours at RT, thereafter staying more or less constant. In samples on wet ice, we found slightly lower concentrations as compared to samples kept on dry ice, and this was also already visible after only 12 hours.

Out of 14 amino acids, concentrations of arginine, glycine, ornithine, phenylalanine, serine and isoleucine increased significantly during pre-storage handling at RT. For phenylalanine and serine, p-values for the time coefficient, temperature coefficient and interaction were significant; for arginine, glycine and ornithine, only those for the temperature coefficient and the interaction were significant; and for isoleucine only the temperature coefficient was significant. The concentration increases became apparent at 12 hours and were clearest for serine (Fig 2). We also found an increase for samples kept on wet ice in contrast to samples on dry ice for arginine, glycine, phenylalanine and serine. The only amino acid with a trend (not significant) to decrease due to storage at RT or on wet ice for 36 hours was glutamine.

All but one of the 9 lysophosphatidylcholines (lysoPC(18:2)) showed highly significant changes due to pre-storage conditions, with p-values for the time coefficient, the temperature coefficient and the interaction between 10^–5^ and 10^–53^; all of these 8 lysophosphatidylcholine concentrations increased clearly at RT (Fig 2). This increase was already evident after 12 hours of handling at RT and continued with longer duration of RT handling. Between samples kept on dry ice and on wet ice, no concentration difference was visible.

In contrast to lysophosphatidylcholines, concentrations of phosphatidylcholines decreased when handled at RT. This was mainly true for the diacylphosphatidylcholines (Fig 2).

In the group of acylalkylphosphatidylcholines we also observed a decrease in concentration during handling at RT, but changes were not as pronounced as for the diacylphosphatidylcholines and were only significant for the temperature coefficient in PC(O-34:1).

### Finding an independent intrinsic marker for pre-analytical quality

**Fig 3:**
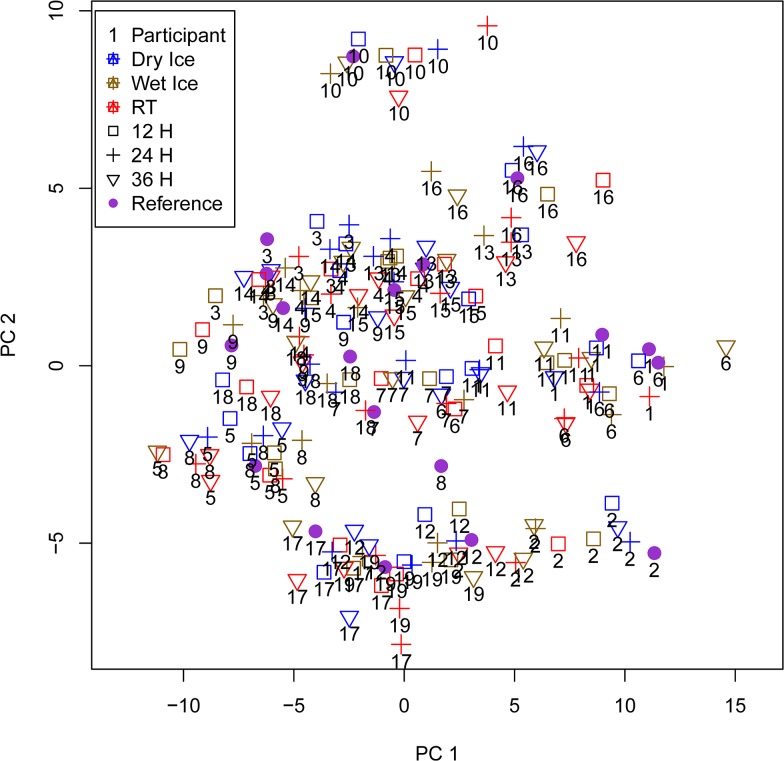
Principal component analysis (PCA) of metabolite concentrations. Projecting all samples from each participant treated by different pre-storage handling conditions on the first two principal components of the PCA based on all metabolite concentrations shows that samples from one participant cluster together in spite of different pre-storage handling conditions. Reference samples (immediately frozen in liquid nitrogen) are depicted by violet circles.

Although changes due to RT temperature handling of our samples were quite pronounced and we found a clear signature of degradation of these samples in the data, individual differences between samples were still much larger and samples could not be differentiated according to handling conditions by principal component analysis (Fig 3). They rather clustered according to the individual donors, showing that inter-individual variability was higher than pre-analytical variability.

As a consequence, cut-off levels for single metabolites, e.g. amino acids and lysophosphatidylcholines, are not sufficient to decide if an individual sample is of ‘good’ or ‘bad’ pre-analytical quality. Therefore, in a second step we calculated metabolite ratios to account for individual differences, thereby focusing on the processes that lead to changes in the metabolite concentrations [16].

We calculated the ratios of all single metabolite concentrations and the p-gain of these ratios as described in the Statistical Analysis section. The p-gain was used as a measure to determine whether a ratio between two metabolite concentrations carries more information than the two corresponding metabolite concentrations alone.

In total we found 235 metabolite ratios with significant p-values and p-gains (S2 Table). The highest p-gains were for amino acid ratios, especially for ratios involving glutamine—which had a downward trend as a single metabolite (not significant)—and those with strong upward trends, e.g. glycine, serine and phenylalanine. Additionally, ratios of phenylalanine to tryptophan and threonine, which were not strongly affected as single metabolites, showed high p-gains.

Ratios between lysophosphatidylcholines and various phosphatidylcholines also displayed high p-gains as was expected due to the increase in lysophosphatidylcholines and decrease in phosphatidylcholines during RT handling of samples.

Our ultimate goal was to make a simple classification model so that with given metabolite ratios from a sample we can determine with high probability whether this sample is of ‘good’ or ‘bad’ pre-analytical quality. To this end, we conducted a two-stage process:

1. We found the variables (metabolite ratios) of greatest importance for classification of the samples by Random Forest (RF) analysis. For this step we used all 235 ‘single’ metabolite ratios with significant p-values and p-gains (S2 Table). Additionally we generated four ‘sum ratios’ of metabolite groups which showed pronounced changes due to RT treatment of samples: total lysophosphatidylcholines/total phosphatidylcholines (tLyso/tPC), tLyso/total diacylphosphatidylcholines (tLyso/tPCaa), tLyso/total acylalkylphosphatidylcholines (tLyso/tPCae) and total amino acids/total acylcarnitines, giving us a total of 239 variables for this step. To make sure that only relevant variables would be selected, we used a stringent definition of ‘good’ and ‘bad’ sample quality in this step: only reference samples were considered ‘good’ and only samples kept for 36 hours at RT were considered ‘bad’.
2. We constructed a model for the classification of our samples using the most important variables found in step 1. For this step we used a less stringent definition of ‘good’ and ‘bad’ sample quality to be able to include more data points and to have a more realistic approach resembling laboratory practice: all samples kept on dry ice and reference samples were considered ‘good’ and samples kept for 24 and 36 hours at RT were considered ‘bad’.

These steps were performed on a training set containing the samples of 12 participants. The samples of the remaining 7 participants were left as a test set for later validation of the resulting models.

**Fig 4:**
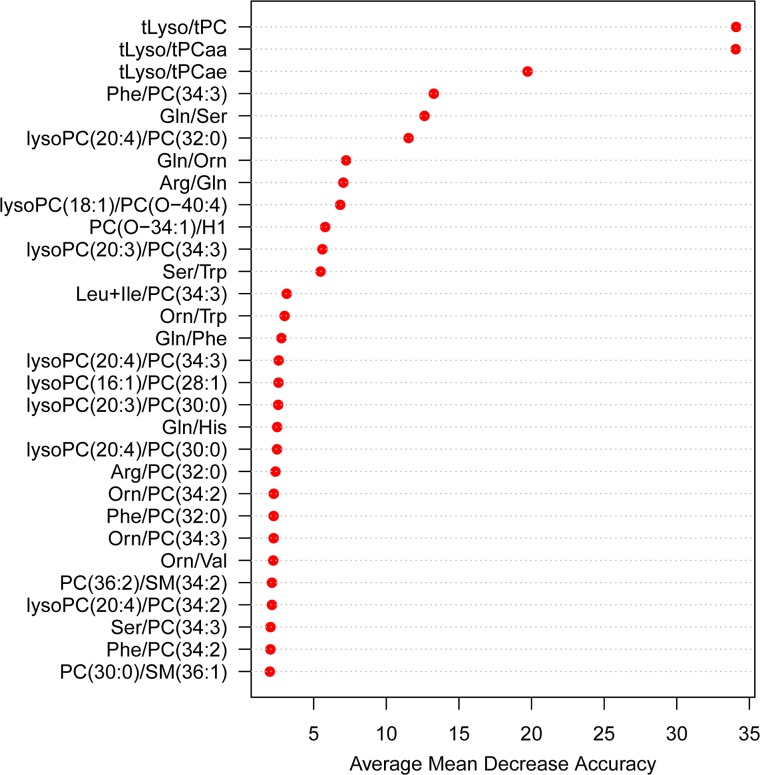
Metabolite ratio importance plot. The importance for each metabolite ratio in classifying samples into ‘good’ or ‘bad’ pre-storage handling conditions was assessed by the average decrease in classification accuracy found in 500 Random Forest analysis iterations (the full names of the metabolites can be found in S1 Table).

The RF analysis led to the ranking of the importance of the variables according to the mean decrease in classification accuracy, seen in Fig 4. The most important variable was a sum ratio, tLyso/tPC.

**Fig 5:**
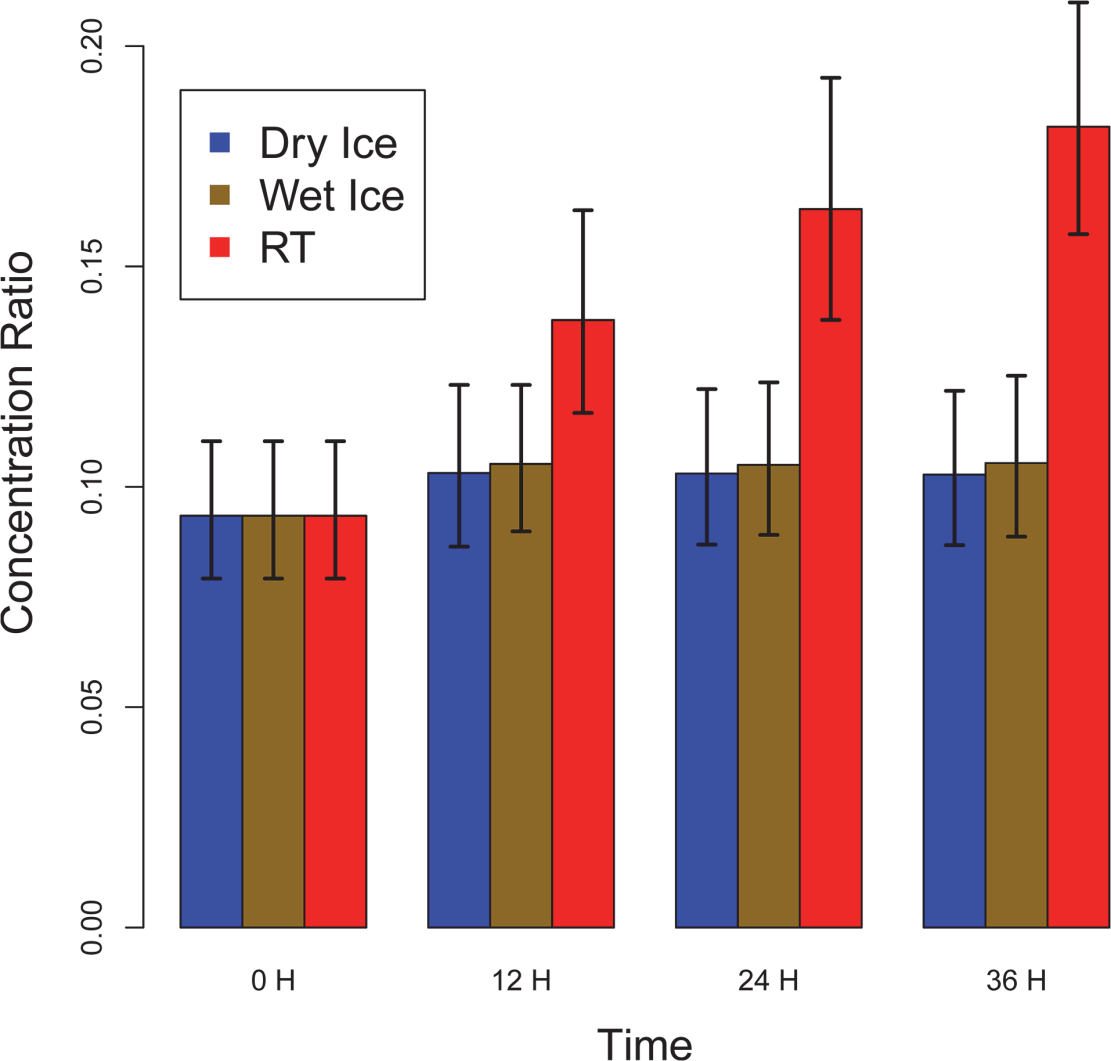
Ratio of total lysophosphatidylcholines to total phosphatidylcholines. The displayed concentration values represent the mean of the samples of all 19 participants at the respective pre-analytical condition. The error bars represent one standard deviation based on the 19 measurements. The rof total lysophosphatidylcholines to total phosphatidylcholines (tLyso/tPC) in samples treated by different pre-storage handling conditions increases with time during RT treatment.

Fig 5 shows a bar plot of this ratio for all handling groups. The two other lysophosphatidylcholine/phosphatidylcholine sum ratios, tLyso/tPCaa and tLyso/tPCae, were the second and third most important variables. Among the ten most important variables were other ratios containing single lysophosphatidylcholines and phosphatidylcholines, showing the importance of this metabolic process for the distinction between ‘good’ and ‘bad’ pre-analytical treatment in our study. The second metabolic process reflected in the top ten variables from step one involves amino acids, with the ratios glutamine/serine (Gln/Ser), glutamine/ornithine (Gln/Orn) and arginine/glutamine (Arg/Gln) ranked fifth, seventh and eighth, respectively.

To construct the classification tree we used the top ten variables from Fig 4. Taking all 10 variables results in a tree with only tLyso/tPC and Gln/Ser as branching points with the following cut-off values: a sample with tLyso/tPC ≥ 0.12 and Gln/Ser ≤ 4.71 was classified as a sample with ‘bad’ pre-analytical quality.

When we classified our samples with this tree based on the non-stringent definition of ‘good ‘ and ‘bad’ we achieved misclassification rates below 5%, namely 0.042 (3/72) for the training set and 0.048 (2/42) for the test set.

To test the robustness of our approach we built further models with varying combinations of the top ten variables. Those models that contained an amino acid variable and a lysophosphatidylcholine/phosphatidylcholine variable gave very similar misclassification rates (S3 Table).

**Fig 6:**
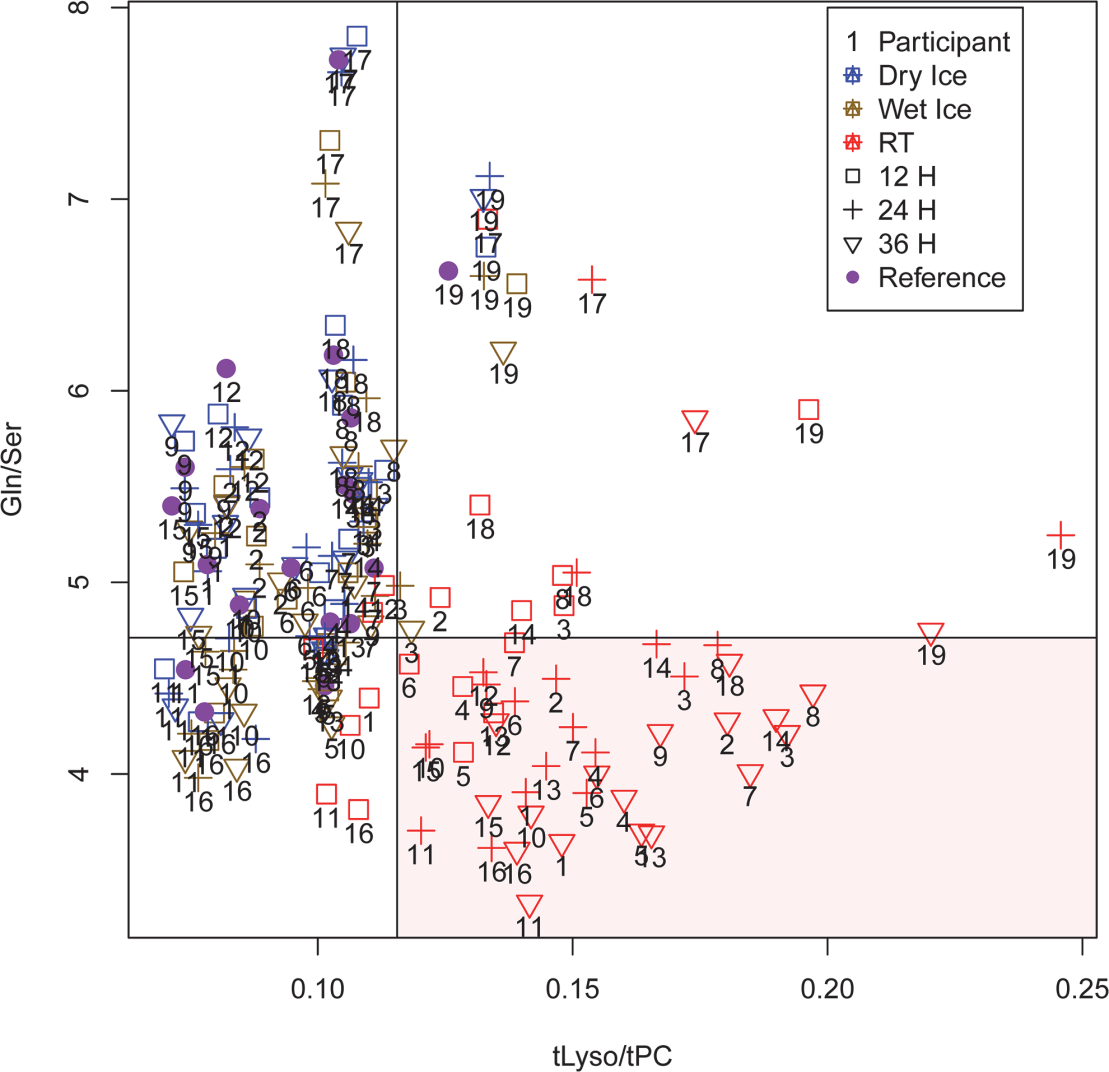
Metabolite concentration ratios for classification of sample quality. Plot of the two metabolite concentration ratios selected by the classification tree out of the 10 most important variables for classification (Fig 4), shown for all samples under different pre-storage handling conditions; cut-off values from the classification tree are shown as horizontal/vertical lines, the red area indicates samples classified as ‘bad’ pre-analytical quality by the classification tree; three samples from the training set (24 and 36 h RT samples of participant Nr. 19, 24 h RT sample of Nr. 18) and two from the test set (24 and 36 h RT of Nr. 17) are wrongly classified as good, giving a misclassification rate of 0.042 on the training set (12 participants) and 0.048 on the test set (7 participants).

We applied the model with tLyso/tPC and Gln/Ser to the remaining handling groups (wet ice samples, 12 hour RT samples). All samples kept on wet ice for 12, 24 and 36 hours were classified as ‘good’. For 12 hour RT samples, 14 were classified as ‘good’ (74%) and 5 as ‘bad’. Fig 6 shows the distribution of all analyzed samples by the tLyso/tPC and Gln/Ser model.

### Stable metabolite concentrations during four freeze-thaw cycles

No significant single metabolite concentration changes were seen in up to four freeze-thaw cycles (S1 Table). Additionally, no metabolite ratio fulfilled the demand of having a significant p-value < 6.3*10^–6^ of the linear regression model and a p-gain > 80010. Only one metabolite ratio, between glycine and threonine, had a significant p-value and a moderate p-gain of 10154 (S2 Table). S1 Fig displays this metabolite ratio for the four freeze-thaw cycles.

For some of the amino acids, namely glycine, methionine, phenylalanine, tryptophan and tyrosine, we observed slight concentration increases with rising numbers of freeze-thaw cycles. In S2 Fig this trend is illustrated for phenylalanine.

## Discussion

We studied concentration changes of 127 metabolites in serum samples due to pre-storage handling conditions as well as due to repeated freeze-thaw cycles.

### Marked concentration changes in metabolites due to pre-storage handling

Our experiment with different pre-storage handling conditions shows pronounced changes in various metabolites due to handling at RT. From the results of the linear regression model we conclude that the important factor for these changes is the temperature at which samples are handled (dry ice vs. wet ice vs. RT) and the interaction between temperature and handling time, but that handling time alone (storage delay) does not have a major effect. The concentration changes at RT occur even though the RT exposure takes place after separation of serum from cells. Boyanton et al [17] analyzed the stability of 24 analytes in plasma and serum stored at RT in contact with cells or after centrifugation and only found changes in these analytes when samples were not separated from cells. Clark et al report changes of only a few percent in fat-soluble vitamins [18] or various plasma analytes [19] when whole blood was stored over several days at RT. Other studies on the influence of pre-centrifugation delay on metabolites [20, 21] attribute the changes they observed to ongoing metabolism due to cells which are still in contact with plasma/serum. But the ongoing changes even after serum separation in our study and also in a study of Bernini [22] on plasma and serum samples kept at RT after centrifugation show that it is not only cells, but probably also enzymes which are secreted or released upon cell damage prior to centrifugation, which account for these changes.

Our results also demonstrate that not all metabolites are equally sensitive to pre-analytical handling conditions. A broad panel of metabolites is therefore necessary to differentiate ‘good’ vs. ‘bad’ handling, as has also been shown by Kamlage et al. in a study on blood and plasma processing [23].

With the targeted metabolomics approach in our study, we find the most pronounced changes in amino acids and phosphatidylcholines. Increases in amino acids can be due to protein degradation processes. This assumption is supported by the fact that in this study increases are especially pronounced for those amino acids which occur with high frequency in proteins (e.g., isoleucine, glycine and serine). In contrast, amino acids with low frequencies in proteins, e.g. tryptophan, do not show marked changes. An exception to this is phenylalanine, which increases clearly in our study. This could be due to the fact that there are only a few degrading pathways for phenylalanine in the cells, which leads to accumulation of phenylalanine.

We find the most striking changes for the group of lysophosphatidylcholines. All of the 9 measured lysophosphatidylcholines—with the single exception of lysoPC(18:2)—increase clearly with handling at RT. In a study conducted by Yang et al [24] with rat plasma kept at 37°C and 4°C for up to 24 hours, the authors also report pronounced increases of up to over 500% in lysophosphatidylcholines, interestingly also with the exception of lysoPC(18:2). At the same time, phosphatidylcholines decrease in our study. Phosphatidylcholines can be hydrolyzed to free fatty acids and lysophosphatidylcholines by phospholipase A2. Phospholipase A2 is a superfamily of enzymes which specifically hydrolyze the sn-2 ester bond of phospholipid substrate, giving a free fatty acid and a lysophospholipid. The family consists of secreted as well as cytosolic and lysosomal enzymes [25]. Therefore it is plausible that phospholipase A2 could be present in serum samples, either as secreted enzyme or due to release upon cell rupture before phase separation. These changes are already visible after 12 hours at RT.

We do find minor changes in some of the metabolites due to storage on wet ice. Changes are less pronounced and, in most of the metabolites, are first visible after 24 hours of post-centrifugation storage delay. Less pronounced and later changes in metabolites due to handling at 4°C as compared to RT have also been found by other authors. Yang et al [16] report clear increases in lysophosphatidylcholines at 4°C, but changes are considerably smaller than at 37°C. Yin [21] finds that the plasma metabolome is stable for up to four hours on ice without plasma separation, whereas changes occur after a room temperature exposure of only 2 hours. A pre-centrifugation delay of 24 hours at 4°C did not influence the proton NMR spectroscopic profile of human serum, whereas the same delay at room temperature had a visible impact on the profile [20]. Bernini et al [22] find slight changes in the first PLS-CA component measured by proton NMR metabolic profiling in uncentrifuged serum and plasma at 4°C and clearly more pronounced changes at 25°C. Our study with serum after phase separation shows that storage on wet ice is an acceptable alternative if immediate freezing of samples is not possible. However, as there are ongoing changes in the samples, the storage on wet ice should be kept as short as possible to keep samples unchanged.

### Independent internal measure for pre-analytical sample quality

Overall, a clear signature of degradation of the RT samples is visible in this data set. This is indicated on the one hand by increases in amino acids, which reflects degradation of proteins, and on the other hand by increases in lysophosphatidylcholines and decreases in phosphatidylcholines, probably caused by the action of phospholipase A2 as described above. Inter-individual differences are still much larger, though, as can be seen by principal component analysis (PCA, Fig 3).

The first components of this PCA do not differentiate between handling conditions or duration of pre-storage delay. Samples rather cluster according to individual donor, indicating that differences in individual metabolite levels are larger than changes due to pre-storage handling conditions. In the study conducted by Yang et al [16] with rat plasma samples, PCA gives a clear distinction between 4° vs. 37°C samples and between samples which are kept at 37°C for shorter vs. longer time intervals. This may be due to the low number of individuals used in Yang´s study (3 rats) and to lower individual variation in metabolic profiles of laboratory animals.

To account for individual differences in our samples we decided to analyze metabolite ratios. Thereby we focus on the metabolic processes that lead to changes in metabolite concentrations and achieve a normalization of individual metabolite levels. The ratios with the highest significances are those between different amino acids, in particular those including glutamine. Glutamine is the only amino acid with a decreasing trend, though not significant as a single metabolite. The decrease is probably due to oxidation of glutamine to glutamate. Unfortunately glutamate was not measured in our panel.

An important result of this study is the development of a model for the distinction between ‘good’ and ‘bad’ pre-analytical quality samples. We used a two-stage process involving Random Forest analysis and a classification tree to find the variables and cut-off values for this distinction. The result was that the ratios of total lysophosphatidylcholine to total phosphatidylcholine and of glutamine to serine can be used to effectively assess prolonged room temperature exposure of a sample. Similar metabolite ratios involving other lysophosphatidylcholine and phosphatidylcholine species and other amino acids, thus representing the same two metabolic processes, gave very similar results.

This study is a first attempt to enable researchers to test their samples for pre-analytical quality before using them for cost- and labour-intensive analysis and is a step towards introducing quality control procedures in biobanking.

Our model classifies all samples kept on wet ice as ‘good’ quality samples. In contrast, 26% of samples kept at RT are already classified as ‘bad’ quality after 12 hours of pre-storage delay. This leads to the clear recommendation to strictly avoid RT handling of serum samples in biobanks or studies.

### Freeze thaw

Especially with older samples, which have not always been aliquoted in appropriate portions right from the beginning of storage, repeated thawing and freezing is an issue in biobanks. We wanted to know if repeated freeze-thaw cycles pose a major problem for the targeted analysis of metabolites. We restricted the number of freeze-thaw cycles analyzed to four, because we think that this is a number that may reflect reality in today’s biobanking.

Freezing/thawing is known to induce conformational changes leading to aggregation or degradation of proteins [26]. In our study, except for slight, non-significant increases in glycine, methionine, tryptophan, phenylalanine and tyrosine, we do not find major changes in concentrations of the 127 metabolites in serum after up to four freeze-thaw cycles. The analysis of metabolite ratios in freeze-thawed samples only yields one ratio with a significant p-value and a moderate p-gain, the ratio between glycine and threonine. The slight increase in phenylalanine and other amino acids may indicate some degradation of proteins which occurs during thawing and refreezing and reflects slightly ongoing metabolism, as found by Wood [27] for plasma arachidonic acid. But from the fact that metabolite ratios are only moderately affected by freeze-thaw cycles, we conclude that no pronounced metabolic processes occur in these samples. Zivkovic [28] also finds few changes in serum lipid composition due to up to three freeze-thaw cycles and Cuhadar et al [29] report good stability of 17 routine chemistry analytes in serum in up to ten freeze-thaw cycles. In contrast, Fliniaux [20] observes a visible impact of freeze-thaw cycles on the metabolic profiles of serum samples, measured by an untargeted proton NMR spectroscopy approach. Therefore, even if changes are not pronounced, caution has to be taken when sensitive molecules are selected for biomarker studies and pre-analytical conditions have to be carefully controlled.

In summary, our data show pronounced changes in various metabolites from different metabolite groups due to pre-storage handling at RT. Increases in concentrations are especially pronounced for amino acids and lysophosphatidylcholines. Individual samples can be classified as ‘good’ or ‘bad’ pre-analytical handling samples by analyzing certain metabolite ratios.

As changes are already clearly visible after 12 hours for a range of metabolites, handling at RT should be strictly avoided in biobanks.

Our model classifies samples handled on wet ice as ‘good’ quality samples. We do find slight changes in some metabolites in those samples, though, which mainly take place after 24 hours of pre-storage delay. Therefore, biobanks should aim to reduce handling time of samples on wet ice and store samples at -80°C or on dry ice as soon as possible.

In contrast, up to four freeze-thaw cycles do not pose a major problem for the metabolites measured in our panel.

## Supporting Information

S1 Fig Change in concentration ratio between glycine and threonine due to up to four freeze-thaw cycles; this metabolite ratio has a significant p-value for the linear regression model for number of freeze-thaw cycles, but only a moderate p-gain of 10154 (< 10*8001). (TIF)

S2 Fig Change in phenylalanine concentration due to up to four freeze-thaw cycles; the p-value for the linear regression model for number of freeze-thaw cycles is not significant. (TIF)

S1 Table List of all 163 metabolites measured with full name (column A), short name (column B) and name used in the Biocrates AbsoluteIDQ p150 kit (column C); the 127 metabolites which passed quality control are shown in black; the 26 metabolites that were excluded due to either having a coefficient of variation (CV) over 25% in 20 pool samples measured on 4 plates (column E) or more than 50% of all measurements below LOD (column F) are shown in grey and the reason for exclusion is highlighted in grey; in column G and H the mean concentration and standard deviation of all reference samples of the 19 participants for the metabolites that passed quality control are given; column I, J and K depict p-values of the linear regression model for time, temperature and interaction (significant ones are shown in bold and highlighted in grey) and column L indicates the direction of the change for the metabolites with significant p-values; column M shows the p-values for the linear regression model for freeze-thaw cycles. (XLS)

S2 Table a) List of metabolite ratios with significant p-values and p-gains higher than 240030 for temperature, time or overall model; b) list of the three metabolite ratios with significant p-values for number of freeze-thaw cycles; none of the three fulfills the demand of a p-gain higher than 80010. (XLS)

S3 Table List of variable combinations out of the top ten variables used for classification trees to divide samples into ‘good’ or ‘bad’ pre-analytical quality and misclassification rates for the training set (12 participants) and the test set (7 participants). (XLS)
